# Supplementary material for: A Humanized Yeast Phenomic Model of Deoxycytidine Kinase to Predict Genetic Buffering of Nucleoside Analog Cytotoxicity
Source: Genes (Basel). 2019 Sep 30;10(10):770. doi: 10.3390/genes10100770 (PMC6826991; doi:10.3390/genes10100770)
Supplement: Supplementary file 1 [file genes-10-00770-s001.zip › Additional_Files/Additional_File7_GOTermHeatmaps.zip!/Additional_File7_GOTermHeatmaps/aminoacyl-tRNA_editing_activity.pdf]

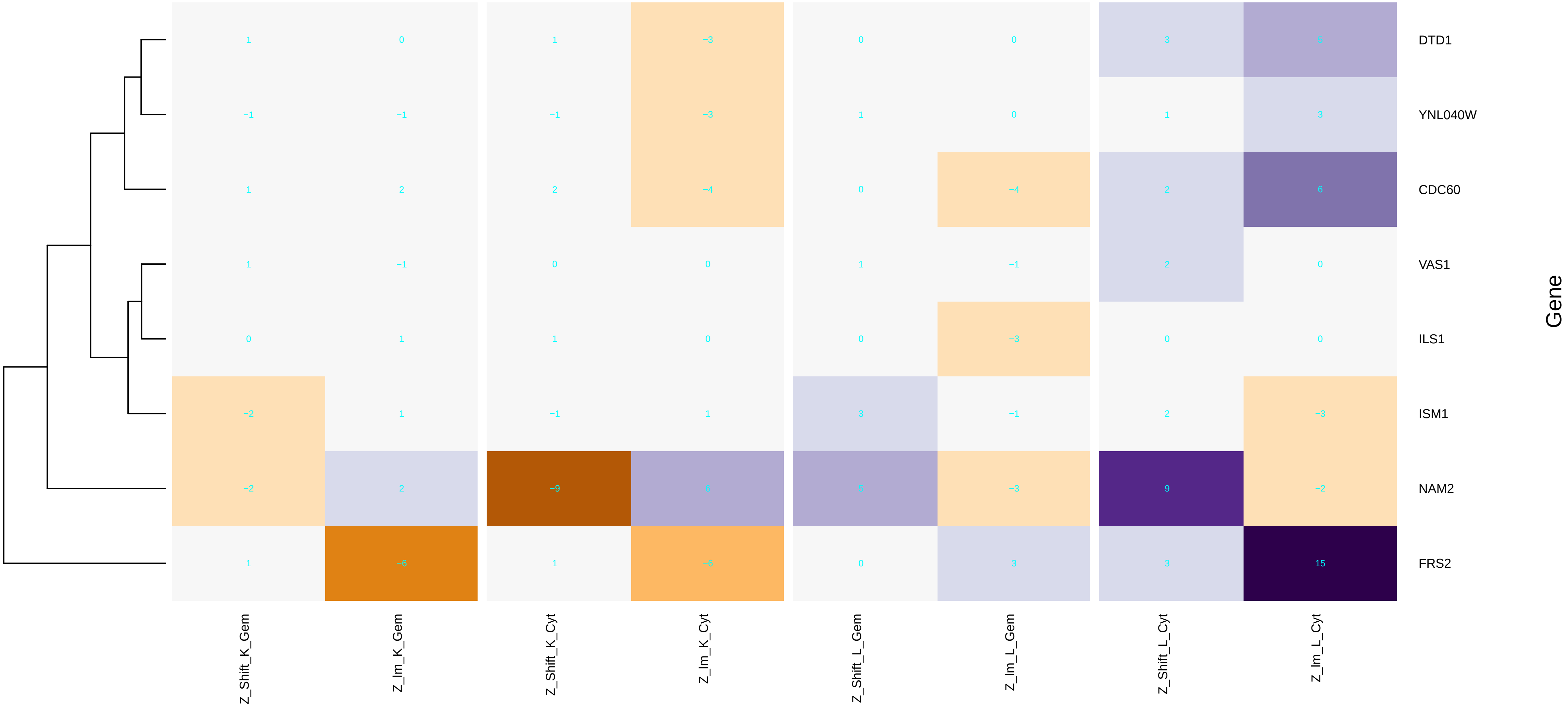
Z_Shift_K_Gem
Z_Im_K_Gem
Z_Shift_K_Cyt
Z_Im_K_Cyt
Z_Shift_L_Gem
Z_Im_L_Gem
Z_Shift_L_Cyt
Z_Im_L_Cyt
DTD1 1 0 1 −3 0 0 3 5
YNL040W −1 −1 −1 −3 1 0 1 3
CDC60 1 2 2 −4 0 −4 2 6
VAS1 1 −1 0 0 1 −1 2 0
ILS1 0 1 1 0 0 −3 0 0
ISM1 −2 1 −1 1 3 −1 2 −3
NAM2 −2 2 −9 6 5 −3 9 −2
FRS2 1 −6 1 −6 0 3 3 15
Gene
